# Unusual findings in secondary hypertension: double orifice mitral associated to aortic coarctation, bicuspid aortic valve, and ventricular septal defect

**DOI:** 10.1186/1755-7682-7-14

**Published:** 2014-04-02

**Authors:** Najat Mouine, Rachida Amri, Mohamed Cherti

**Affiliations:** 1 Department of Cardiology B, IBN SINA Hospital, Mohamed V University Souissi, Rabat, Morocco

**Keywords:** Secondary hypertension, Aortic coarctation, Bicuspid aortic valve, Double orifice mitral, Ventricular septal defect

## Abstract

Double orifice mitral valve is a rare congenital anomaly presenting as the division of the mitral orifice into two anatomically distinct orifices, it is most often associated with other congenital heart defects such as left-sided obstructive lesions, ventricular septal defects or aortic coarctation. We report the case of a 15 year’s old boy, admitted for arterial hypertension, auscultation revealed a rude aortic systolic murmur. Femoral pulses were weak. Owing to the suspicion of aortic coarctation, transthoracic echocardiography was performed, the aortic coarctation with dilation of the aorta proximal to the stenosis was confirmed and bicuspid aortic valve was found with good function. The mitral valve was dysmorphic, having two orifices; it was divided into 2 separate valve orifices by a fibrous bridge. No mitral or aortic regurgitation was documented by color Doppler flow imaging. The left ventricular ejection fraction was normal. There was a small peri membranous ventricular septal defect with left to right shunt. Owing to the severity of the aortic coarctation and taking into account the anatomy and characteristics of the patient, he was made a surgical correction of aortic coarctation with good outcome.

## Introduction

Double orifice mitral valve is a rare congenital anomaly presenting as the division of the mitral orifice into two anatomically distinct orifices, it is most often associated with other congenital heart disease (CHD) such as left-sided obstructive lesions, ventricular septal defects or aortic coarctation.

## Case report

**Figure 1:**
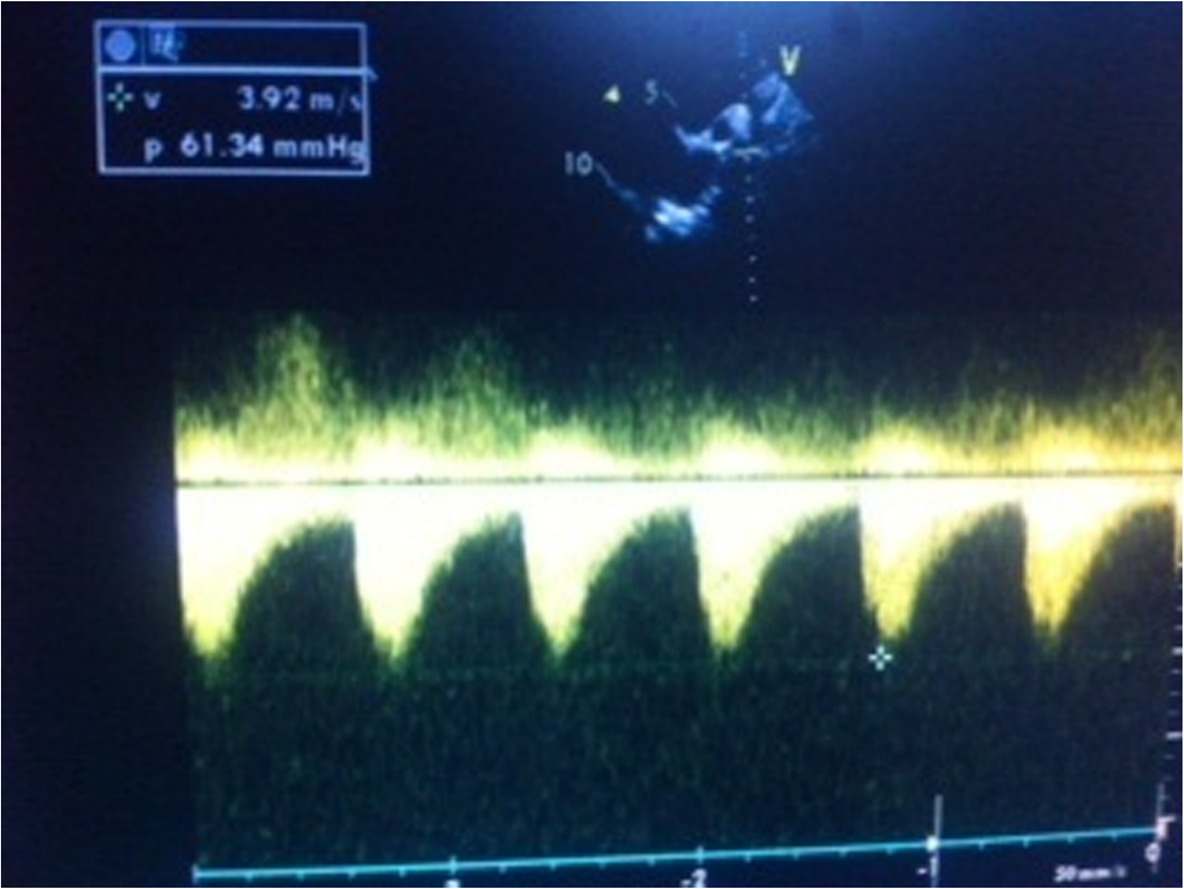
Echocardiography supra sternal view shows aortic coarctation with dilation of the aortic segment proximal to the stenosis and acceleration of the flow.

**Figure 2:**
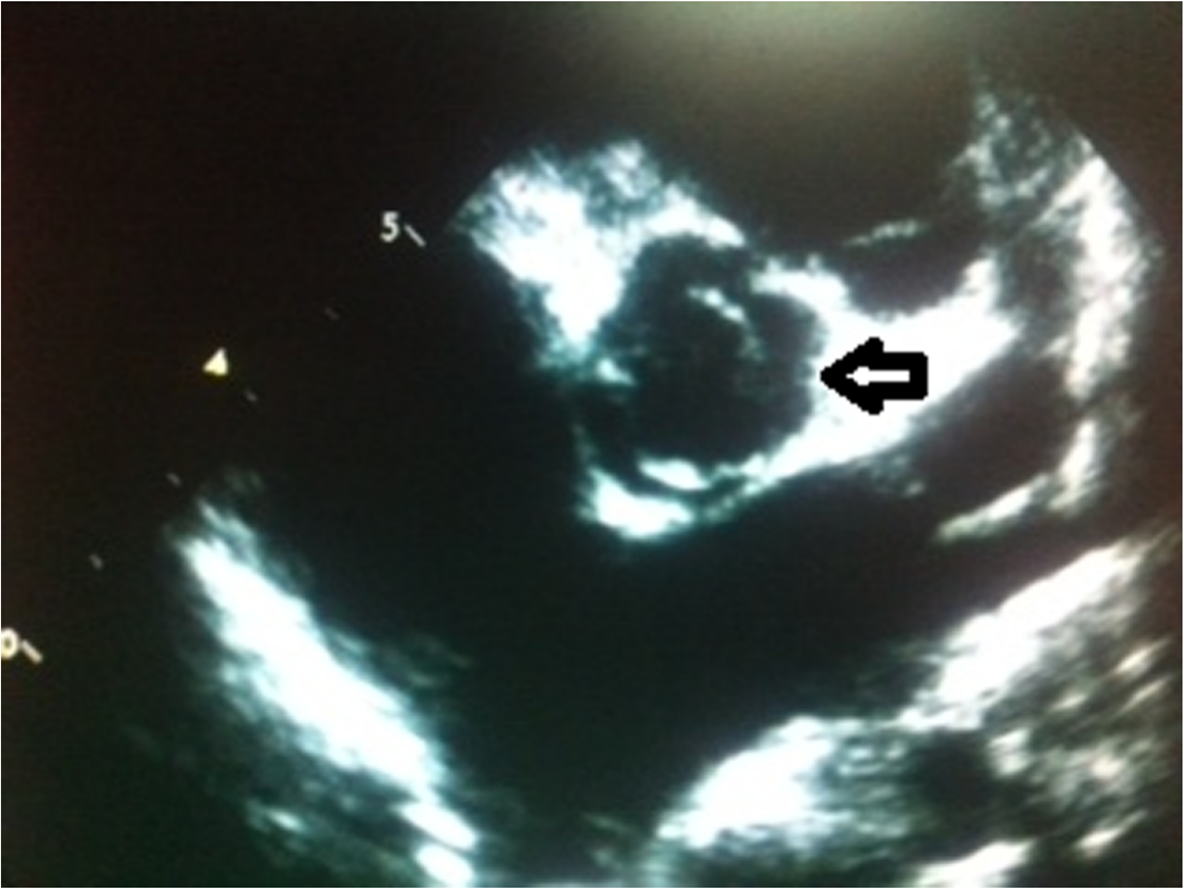
Echocardiography short axis parasternal view showed bicuspid aortic valve.

A 15 year’s old boy, admitted for arterial hypertension ^(BP: 160/90mmHg)^, auscultation revealed a rude aortic systolic murmur. Femoral pulses were weak, biological assessment was negative. Owing to the suspicion of aortic coarctation, transthoracic echocardiography was performed, the aortic coarctation with dilation of the aorta proximal to the stenosis was confirmed and bicuspid aortic valve was found with good function [Figures 1 and 2].

**Figure 3:**
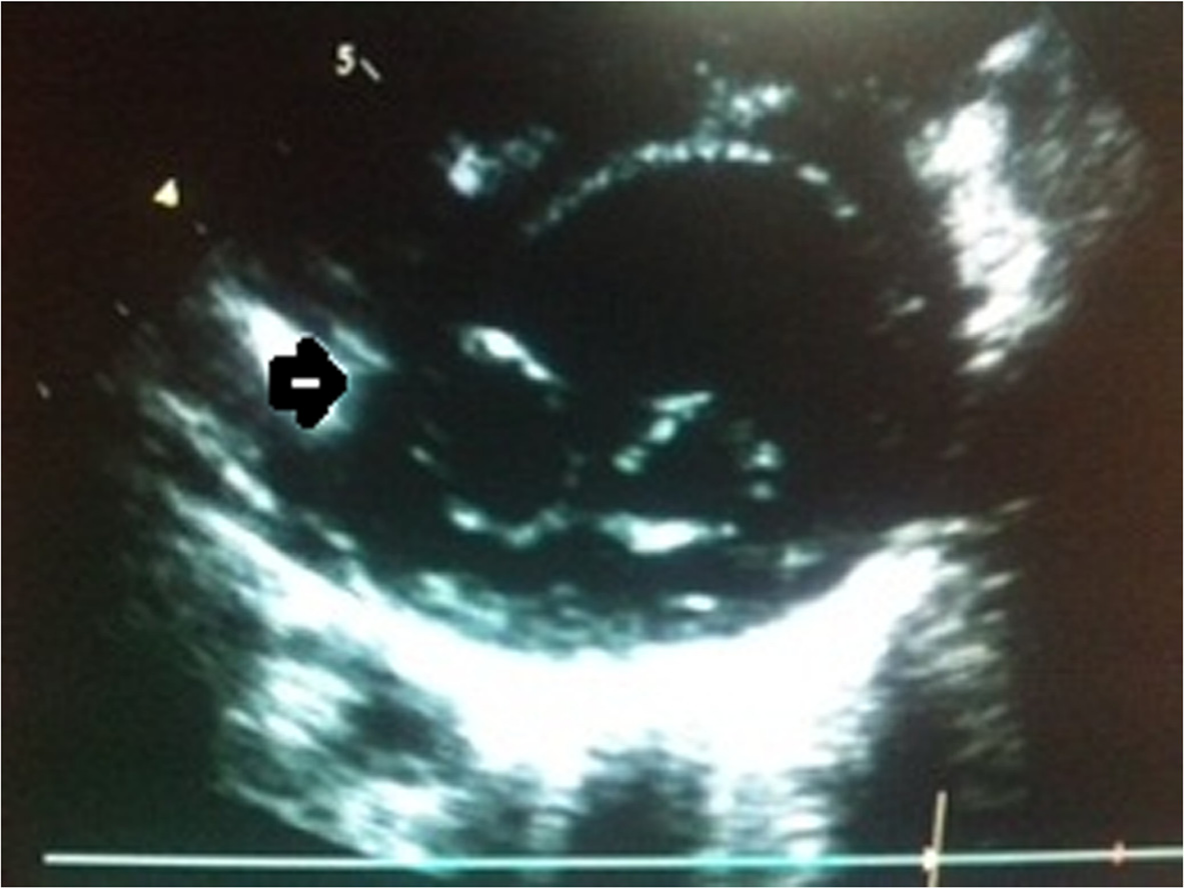
Echocardiography short axis parasternal view showed double orifice mitral valve.

The mitral valve was dysmorphic, having two orifices; it was divided into 2 separate valve orifices by a fibrous bridge [Figure 3]. Color Doppler flow imaging of the mitral valve showed 2 separate envelopes of anterograde flow into the left ventricle through the double orifices during diastole. No mitral or aortic regurgitation was documented by color Doppler flow imaging. The left ventricular ejection fraction was normal. There was a small peri membranous ventricular septal defect with left to right shunt (Vmax = 6,29 m/s).

Owing to the severity of the aortic coarctation and taking into account the anatomy and characteristics of the patient, he was made a surgical correction of aortic coarctation with good outcome.

## Discussion

First case is described in 1876 by Greenfield [1], double orifice mitral valve is an uncommon anomaly which, as its name indicates, has a single mitral annulus and opens into the left ventricle through two orifices. The normal mitral valve consists of a central orifice located between a sail-like anterior leaflet and a C-shaped posterior leaflet. The anterior mitral leaflet occupies roughly one-third of the annular circumference and the posterior leaflet occupies roughly the remaining two-thirds of the annular circumference. In a double orifice mitral valve; however, abnormal tissue divides the mitral orifice into 2 parts [2]. Mitral orifice are unequal in size, with the smaller orifice directed towards the anterolateral commissure or the posteromedial commissure. The mitral valve can function reasonably well in about 50% of patients. In the other 50%, it can cause clinically significant mitral stenosis or mitral regurgitation. The commonly associated lesion is atrioventricular septal defect, aortic coarctation, interrupted aortic arch, patent ductus arteriosus, primum atrial septal defect, tetralogy of Fallot, and Ebstein anomaly [3,4]. In our case, double orifice mitral valve is associated to ventricular septal defect, aortic coarctation and bicuspid aortic valve. Rosenberg et al. reported 25% of patients with double orifice mitral valve have partial persistent atrio ventricular canal [5]. Bibhuti Das et al. found that various abnormalities along with double orifice mitral valve were left-sided obstructive lesions, ventricular septal defect; anomalies of the tricuspid valve [2] like our case, there was aortic coarctation, ventricular septal defect and biscuspid aortic valve as an associated anomaly. Management depends on the type and severity of mitral valve dysfunction. Isolated double orifice mitral valve causing neither obstruction nor regurgitation needs no active intervention. Surgical intervention is necessary when stenosis or incompetence is severe or if repair of an associated cardiac lesion is needed, in our case, the patient had only surgical correction of aortic coarctation with good outcome. Long-term follow-up is necessary to detect subsequent abnormal hemodynamics or complications.

## Consent

A written informed consent was obtained from the patient for the publication of this paper and the accompanying images.

## Competing interests

The authors declare that they have no competing interest.

## Authors’ contributions

RA participated in the sequence alignment and drafted the manuscript. MC participated in the sequence alignment and drafted the manuscript. All authors read and approved the final manuscript.
